# Incidence and geographic mapping of meniscal tears in acute ACL injuries with meticulous posterior arthroscopic evaluation: longitudinal tears at the posterior menisco-capsular junction are the most common tear pattern in acute ACL injuries

**DOI:** 10.1186/s43019-026-00301-z

**Published:** 2026-01-22

**Authors:** Dong Hwi Kim, Do Kyung Lee

**Affiliations:** 1 Department of Orthopedic Surgery, Chosun University Hospital, Chosun University School of Medicine, Gwangju, Korea; 2 Department of Orthopedic Surgery, Samsung Changwon Hospital, Sungkyunkwan University School of Medicine, Changwon, Korea

**Keywords:** Meniscal injuries, Incidence, Anterior cruciate ligament, Menisco-capsular junction, Longitudinal tears, Acute ACL injuries

## Abstract

**Background:**

Meniscal injuries are commonly observed in acute anterior cruciate ligament (ACL) injuries, with varying reports on the incidence and location of meniscal tears. In particular, the incidence of posterior menisco-capsular tears may have been underestimated in previous literature owing to the technical challenges associated with posterior arthroscopic evaluation. This study aimed to determine the true incidence and location of posterior menisco-capsular junction tears in patients undergoing anterior cruciate ligament reconstruction (ACLR) up to 6 weeks of injury, hypothesizing a higher incidence than previously reported.

**Methods:**

A retrospective analysis was conducted on patients who underwent primary ACLR between July 2015 and January 2023, including a total of 139 patients. Arthroscopic findings and surgical records were reviewed, and the posteromedial and posterolateral joint spaces were evaluated via intercondylar notch view with a 70-degree arthroscope or via a posteromedial/posterolateral portal as a viewing portal to confirm posterior menisco-capsular junctional tears in all patients. The Cooper’s classification system was used to document tear locations, and radial and longitudinal tear components were analyzed.

**Results:**

Meniscal tears were observed in 104 patients (74.8%). Medial meniscus tears occurred in 63 patients (45.3%), while lateral meniscus tears were found in 78 patients (56.1%). Bilateral meniscus tears were identified in 37 patients (26.6%). Radial tears were predominantly observed in the lateral meniscus (22.3%), with a significant portion being lateral meniscus root tears (15.1%). Longitudinal tears, particularly at the menisco-capsular junction, were the most common tear pattern, occurring in 64.7% of patients.The most commonly involved tear sites were Zone A0 (39.6%) and Zone A1 (42.4%) in the medial meniscus, and Zone F0 (36.7%) and Zone F1 (38.1%) in the lateral meniscus.

**Conclusions:**

Longitudinal tears at the menisco-capsular junction are the most common tear pattern in acute ACL injuries, revealing a higher incidence than previously reported. In acute ACL injuries, Zones A0–A1 and F0–F1 should be meticulously evaluated using an intercondylar notch view with a 70-degree arthroscope or via a posteromedial/posterolateral portal as a viewing portal.

## Background

The primary goal of anterior cruciate ligament reconstruction (ACLR) is to restore normal knee kinematics and prevent the progression of osteoarthritis [1, 2]. Residual anterolateral rotatory instability has been identified as a key factor contributing to osteoarthritis progression [3] Despite significant advancements in surgical techniques such as anatomical ACLR, double bundle reconstruction, and ALL reconstruction or tenodesis, it has been reported that anterolateral rotatory instability remains in 21–37% of patients even after ACLR [4], posing a significant risk for osteoarthritis development.

Recently, there has been growing interest in secondary stabilizers that contribute to improving residual anterolateral rotatory instability [5]. Meniscal injuries have increasingly been recognized as critical contributors [6, 7], with recent studies suggesting that meniscal tears significantly impact anterolateral rotatory instability [7–14]. In addition, some clinical studies have shown that aggressive repair of meniscal tears during ACLR could improve residual rotational instability [13, 15]. In particular, medial meniscus tears at the menisco-capsular junction, known as ramp lesions, have gained attention as potential secondary stabilizers for anterolateral rotatory instability [5, 12, 16]. Previous magnetic resonance imaging (MRI) studies have reported that ramp lesions may be present in approximately 30% of cases, even when MRI findings appear normal [17]. Sonnory-Cottet et al. also emphasized the importance of meticulous posterior arthroscopic evaluation for the accurate diagnosis of ramp lesions [18].

The reported incidence of meniscal tears varies from 44.4 to 65% [19–23], while the incidence of menisco-capsular junctional tears ranges between 15 and 40% [16, 18, 24–26]. In addition, there are conflicting findings regarding the location and tear patterns of meniscal injuries in different studies [19–21, 24]. These inconsistencies may be attributed to differences in the timing of surgical intervention for ACL rupture and the extent to which posterior arthroscopic evaluation was meticulously performed among researchers.

Considering these factors, we hypothesized that the actual incidence of menisco-capsular junction tears is higher than previously reported. Therefore, this study was designed to determine the true incidence of posterior menisco-capsular junction tears occurring in the early phase of ACL injury by conducting a thorough posterior arthroscopic examination in all patients who underwent ACLR up to 6 weeks of injury.

## Methods

### Patient selection

A retrospective analysis was conducted on patients who underwent primary ACLR by two surgeons at both institutes between July 2015 and January 2023, including 131 cases from Chosun University Hospital and 8 cases from Samsung Changwon Hospital. This study was approved by the Institutional Review Board (IRB Number SCMC 2024-06-011). The study aimed to investigate the incidence of meniscal injuries in acute ACL ruptures. Only patients who underwent ACLR up to 6 weeks after injury were included. Medical records and arthroscopic findings were reviewed retrospectively to determine the incidence of meniscal tears. Patients who underwent ACLR beyond 6 weeks after trauma or exhibited radiologic findings of chronic ACL insufficiency (*n* = 62) were excluded on the basis of medical records, MRI, and arthroscopic findings.

Patients meeting any of the following criteria were excluded from the study: (a) Patients lacking arthroscopic images (*n* = 2), making retrospective analysis of meniscal injuries impossible. (b) Patients with multi-ligament injuries or fractures (*n* = 37), in which the injury mechanism differs from primary ACL rupture. (c) Patients with Kellgren–Lawrence grade II or higher osteoarthritic changes identified on long bone scanogram (*n* = 4) or a history of ipsilateral knee surgery (*n* = 5), making it difficult to determine whether the meniscal tear occurred simultaneously with the ACL rupture.

**Fig. 1:**
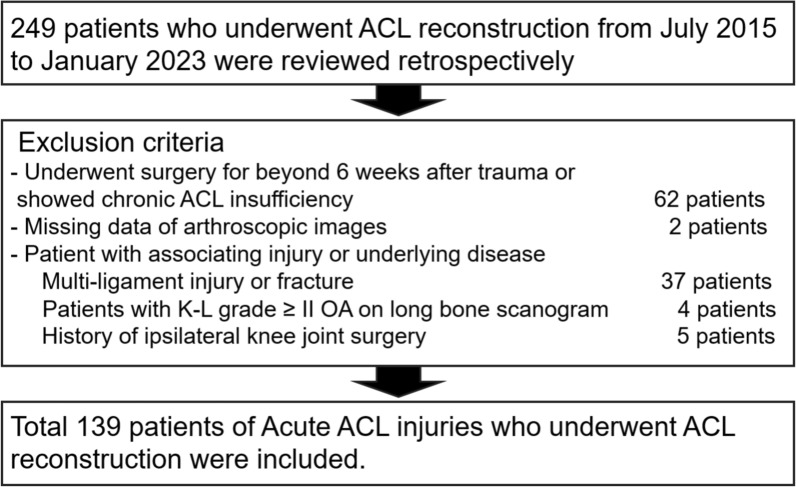
Flow chart

A total of 139 patients were enrolled in this retrospective study (Fig. 1). The demographic characteristics are summarized in Table 1.

**Table 1:** Demographic characteristics of patients with acute ACL injury

Age (year)	30.5 ± 14.7 (14–59 year)
Gender	
Male	104 (74.8%)
Female	35 (25.2%)
Side of injured knee	
Right	76 (54.7%)
Left	63 (45.3%)
Duration from trauma to ACL reconstruction (days)	16.4 ± 10 (1–42 days)
Injury mechanism	
Noncontact injury	119 (85.6%)
Contact injury or direct trauma	20 (14.4%)
Cause of injury	
Twisting	90 (64.7%)
Slip down	14 (10.1%)
Jump and landing	23 (16.6%)
Miscellaneous	12 (8.6%)

### Diagnostic and surgical procedure

Prior to primary ACL reconstruction (ACLR), the Lachman test and Pivot shift test were performed on patients with suspected ACL tears on the basis of MRI findings. Patients exhibiting grade I or higher in the anterior drawer test, Lachman test, or Pivot shift test were diagnosed with ACL dysfunction and subsequently underwent primary ACLR.

In all patients undergoing ACLR, a diagnostic arthroscopic examination was performed by two surgeons using either an anterolateral or anteromedial portal to confirm meniscal tears. Meniscal tears at the posteromedial and posterolateral menisco-capsular junctions were identified in all patients using a 70-degree arthroscope passed through the intercondylar notch or by creating an additional posteromedial or posterolateral portal as a viewing portal with a 30-degree arthroscope. When the meniscal tear was deemed unstable, meniscal repair was performed concurrently with ACLR.

### Data collection

A single surgeon retrospectively reviewed the surgical records of all patients. The type and location of meniscal tears were reconfirmed by reviewing arthroscopic images. Meniscal tear locations were documented using Cooper’s classification system [19]. In addition, meniscal tears were categorized into radial and longitudinal tear components. Radial or oblique tears occurring within approximately 1 cm of the meniscal root bony insertion were defined as root tears [24]. The incidence of root tear was recorded as a subgroup within the radial tear component. Bucket-handle tear was defined as a longitudinal tear that continuously involves two adjacent zones according to cooper’s classification and is considered unstable upon arthroscopic probing if the tear showed displacement into intercondylar notch or around the femoral condyle [27]. The incidence of bucket-handle tears was recorded as a subgroup of the longitudinal tear component. As our study aimed to quantify tears occurring in association with ACL injury, degenerative horizontal tears that could result from osteoarthritis or degenerative changes were intentionally excluded.

All available preoperative MRI scans were reviewed. Because some MRI data were missing or lost, analyses were conducted in 96 patients. Meniscal tears were evaluated according to their location (medial or lateral meniscus) and classified on MRI as radial or longitudinal tears. Degenerative horizontal tears, which are not directly associated with ACL rupture, were intentionally excluded from the analysis.

### Statistical analysis

During the incidence analysis, it was observed that the incidence of lateral meniscus tears at the menisco-capsular junction was lower in patients with lateral meniscus root tears. To determine the statistical significance of this difference, patients were divided into two groups: those with lateral meniscus root tears and those without. The incidence of meniscal tears identified on arthroscopy and MRI was compared using the McNemar test for paired binary data, as both evaluations were performed in the same patients. Subsequently, based on the arthroscopic findings that confirmed the actual presence of meniscal tears, the corresponding MRI findings were matched to calculate the sensitivity and specificity of MRI in detecting meniscal tears. The incidence of menisco-capsular junction tears was compared using a chi-square test, with statistical significance defined as *p* < 0.05. All statistical analyses were performed using SPSS Version 20.0 (IBM Corp., Armonk, NY, USA).

## Results

### The incidence of meniscal tear

**Fig. 2:**
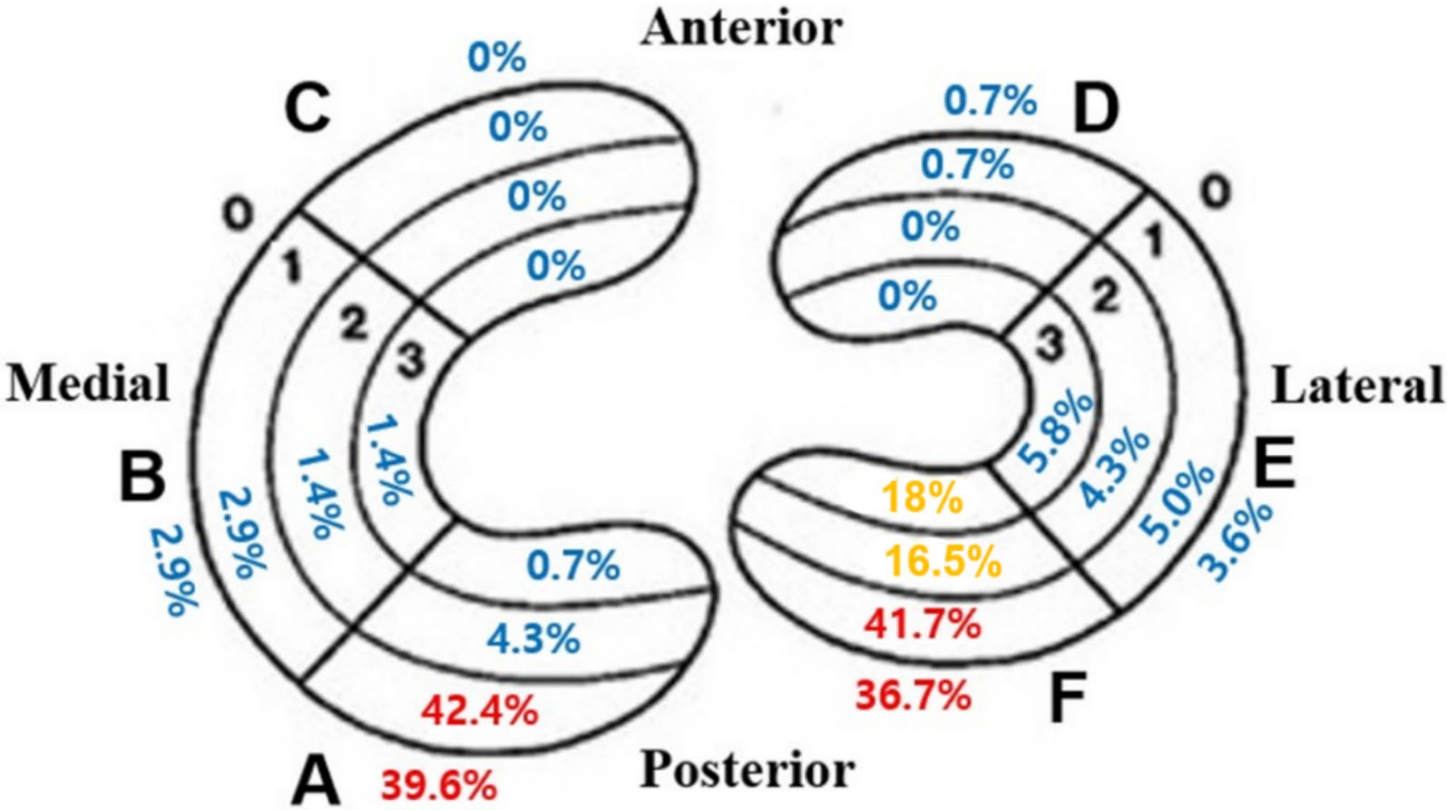
The incidence of meniscal tear in acute ACL rupture according to Cooper’s classification Zones A0–A1 and F0–F1 were the most common locations for meniscal tears

Among patients who underwent ACL reconstruction within 6 weeks of trauma, meniscal tears were observed in 104 of 139 patients (74.8%). Medial meniscus tears were identified in 63 patients (45.3%), while lateral meniscus tears were found in 78 patients (56.1%). Bilateral meniscus tears were present in 37 patients (26.6%). The distribution of meniscal tears is illustrated in Fig. 2, with Zones A0–A1 and F0–F1 being the most common tear sites.

### Incidence of radial tear component

**Fig. 3:**
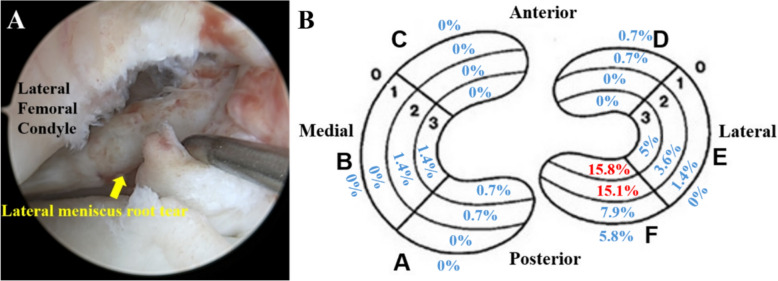
Incidence of radial tears in acute anterior cruciate ligament (ACL) rupture according to Cooper’s classification **A** Arthroscopic view of the right knee obtained using a 30-degree scope with the anterolateral portal as the viewing portal. **B** Zones F2–F3 were the most common locations for radial tears. The lateral meniscus root tear was the most common radial tear pattern in acute ACL rupture

Radial tears were observed in 31 patients (22.3%), with 3 (2.2%) occurring in the medial meniscus, 29 (20.9%) in the lateral meniscus, and 1 (0.7%) in both menisci. Among patients with radial tears, 93% (29/31) were located in the lateral meniscus, whereas 9.6% (3/31) occurred in the medial meniscus, indicating that radial tears predominantly affect the lateral meniscus. In addition, 21 patients (15.1%) exhibited lateral meniscus root tears, accounting for 72.4% (21/29) of all lateral meniscus radial tears. The distribution of radial tears is illustrated in Fig. 3, with Zones F2–F3 identified as the most common sites. Among patients with lateral meniscus root tears, 8 (38.1%) exhibited complete radial tears involving Zones F0 through F3, while 13 (61.9%) exhibited partial tears confined to Zones F2 through F3, with Zone F1 remaining intact.

**Fig. 4:**
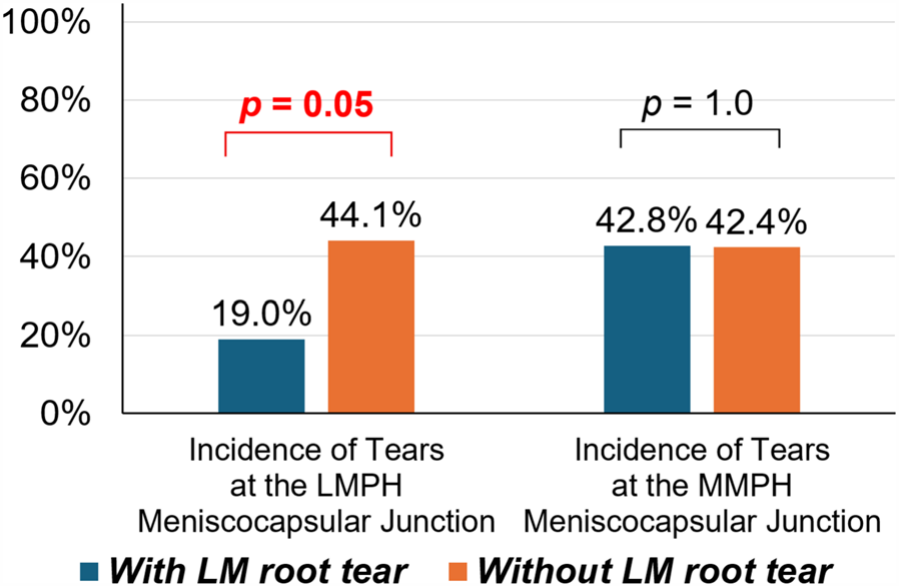
The incidence of meniscal tears at the posterior menisco-capsular junction on the basis of presence of a lateral meniscus root tear 19% 4/21) had a lateral meniscus longitudinal tear at the menisco-capsular junction, which was significantly lower than the incidence in patients without a lateral meniscus root tear (44.1%, 52/118, Odds Ratio: 3.3, *p* = 0.05). Meanwhile, 42.8% (9/21) had a ramp lesion (medial meniscus longitudinal tear at the menisco-capsular junction), with no statistically significant difference compared with patients without a lateral meniscus root tear (42.4%, 50/118, *p* = 1.0)

Among 21 patients with lateral meniscus root tears, 19% (4/21) had a lateral meniscus longitudinal tear at the menisco-capsular junction, which was significantly lower than the incidence observed in patients without a lateral meniscus root tear (44.1%, 52/118) (Odds Ratio: 3.3, *p* = 0.05, Fig. 4). Meanwhile, 42.8% (9/21) had a ramp lesion (medial meniscus longitudinal tear at the menisco-capsular junction). However, there was no statistically significant difference compared with the incidence in patients without a lateral meniscus root tear (42.4%, 50/118) (*p* = 1.0, Fig. 4).

### Incidence of longitudinal tear component

**Fig. 5:**
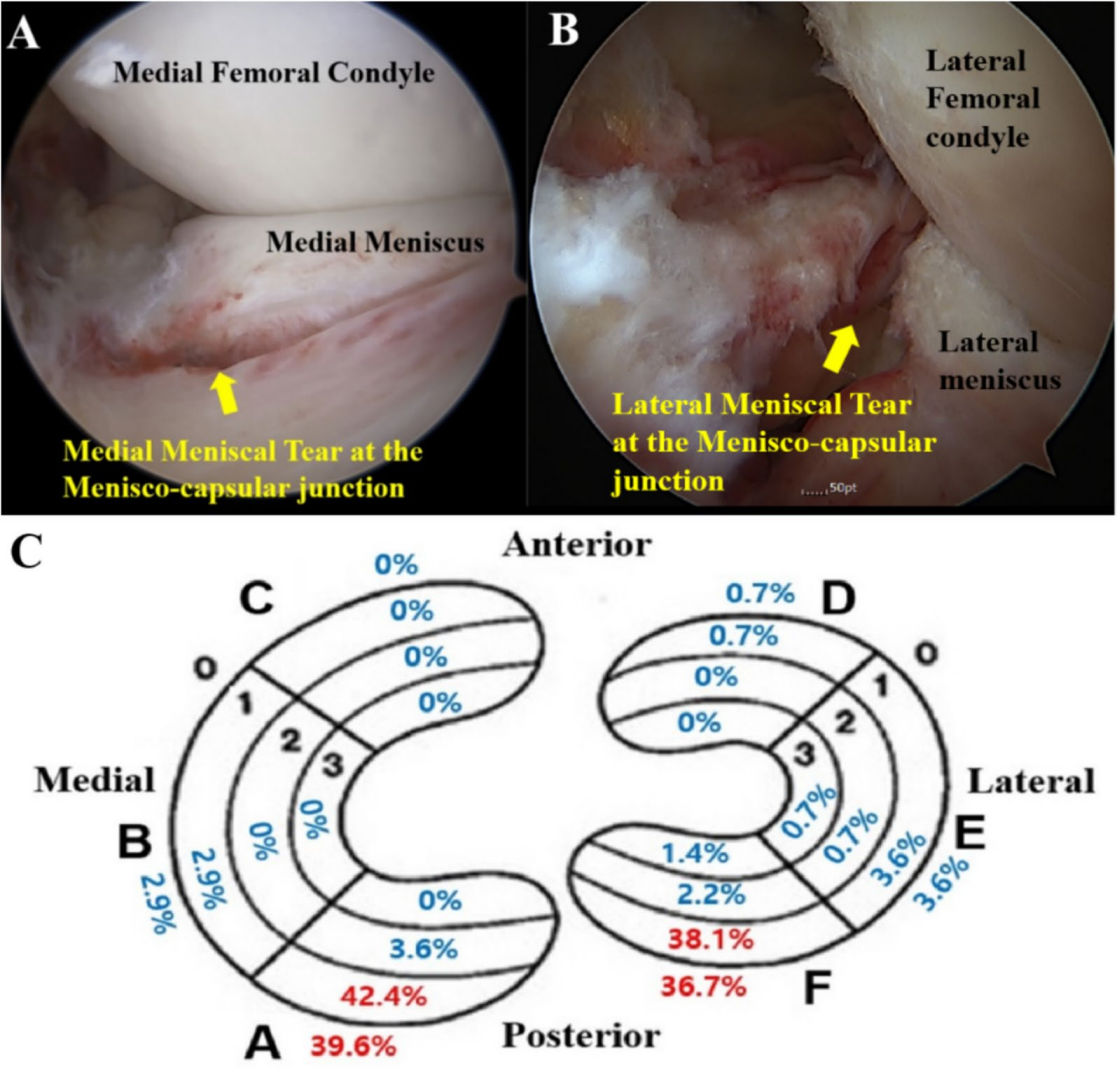
Incidence of longitudinal tear in acute ACL rupture according to Cooper’s classification **A** Arthroscopic view of the left knee obtained using a 30-degree scope with the posteromedial portal as the viewing portal. **B** Arthroscopic view of the left knee obtained with a 70-degree scope through the anteromedial portal, passing across the intercondylar notch. **C** Zones A0–A1 and F0–F1 were the most common locations for longitudinal tears. Longitudinal tears at the menisco-capsular junction were the most common tear pattern in acute ACL rupture

Longitudinal tears were observed in 90 patients (64.7%). Medial longitudinal tears occurred in 59 patients (42.4%), while lateral longitudinal tears were found in 56 patients (40.3%). Concurrent medial and lateral longitudinal tears were present in 25 patients (18%). Longitudinal tears at the menisco-capsular junction were the most common tear pattern in acute ACL rupture. Medial bucket-handle tears occurred in four patients (2.9%), while lateral bucket-handle tears were observed in five patients (3.6%). The distribution of longitudinal tears is illustrated in Fig. 5, with Zones A0–A1 and F0–F1 identified as the most frequent tear sites.

### Comparison of meniscal tear detection between arthroscopy and MRI

**Fig. 6:**
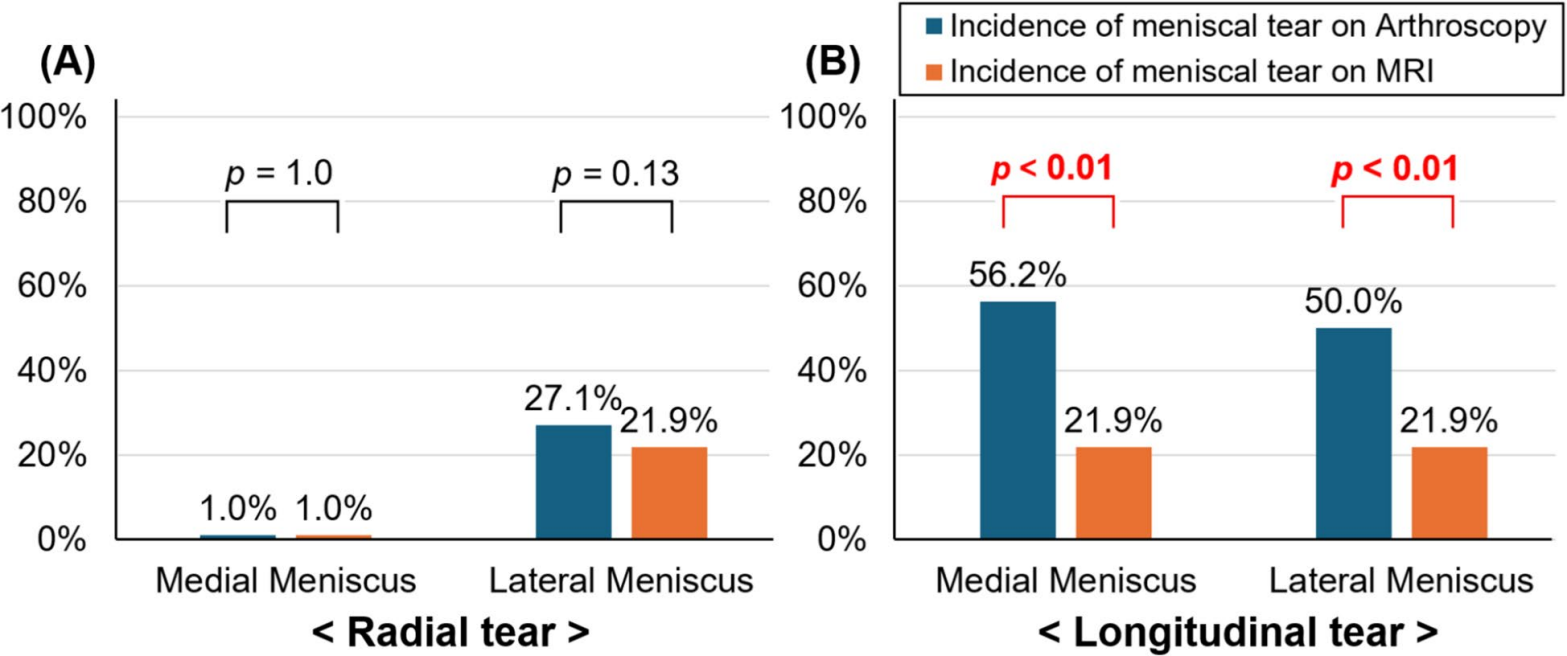
Comparison of the incidence of meniscal tear types between arthroscopy and MRI. **A** For radial tears, the incidence in the medial and lateral menisci was 1.0% (1/96) and 27.1% (26/96) on arthroscopy and 1.0% (1/96) and 21.9% (21/96) on MRI, respectively, showing no significant difference (*p* = 1.0, *p* = 0.13). **B** In contrast, longitudinal tears were observed in 56.2% (54/96) and 50.0% (48/96) of medial and lateral menisci on arthroscopy, but only 21.9% (21/96) for both on MRI, demonstrating significant differences (*p* < 0.01 for both)

Among the patients for whom arthroscopic and MRI findings could be matched, the incidence of radial tears of the medial and lateral menisci was 1.0% (1/96) and 27.1% (26/96), respectively, on arthroscopy, and 1.0% (1/96) and 21.9% (21/96), respectively, on MRI. No statistically significant differences were observed (*p* = 1.0 and *p* = 0.13, respectively). In contrast, the incidence of longitudinal tears of the medial and lateral menisci was 56.2% (54/96) and 50.0% (48/96), respectively, on arthroscopy, but only 21.9% (21/96) for both menisci on MRI, showing statistically significant differences (*p* < 0.01 for both; Fig. 6).

Regarding diagnostic accuracy, the sensitivity and specificity for detecting radial tears were 100% and 100% for the medial meniscus, and 76.9% and 98.6% for the lateral meniscus, respectively. For longitudinal tears, the sensitivity and specificity were 37.7% and 97.6% for the medial meniscus, and 34.0% and 91.0% for the lateral meniscus, respectively.

## Discussion

In this study, concurrent medial or lateral meniscal tears were observed in 74.8% of patients with acute ACL ruptures. The incidence of medial and lateral meniscus tears was 45.3% and 56.1%, respectively, while 26.6% of patients exhibited bilateral meniscal tears. According to Cooper’s classification, the most frequently injured regions of the meniscus were Zone A and Zone F, corresponding to the posterior horns of the medial and lateral meniscus. Radial tear analysis revealed that radial tears predominantly occurred in the lateral meniscus, with the most common pattern being lateral meniscus root tears involving Zones F2–F3, observed in 15.1% of patients. Longitudinal tear analysis indicated that the most common pattern of longitudinal tears occurred at the menisco-capsular junction, specifically in Zones A0–A1 and F0–F1. These tears were identified in 42.4% of medial meniscus tears and 40.3% of lateral meniscus tears.

**Fig. 7:**
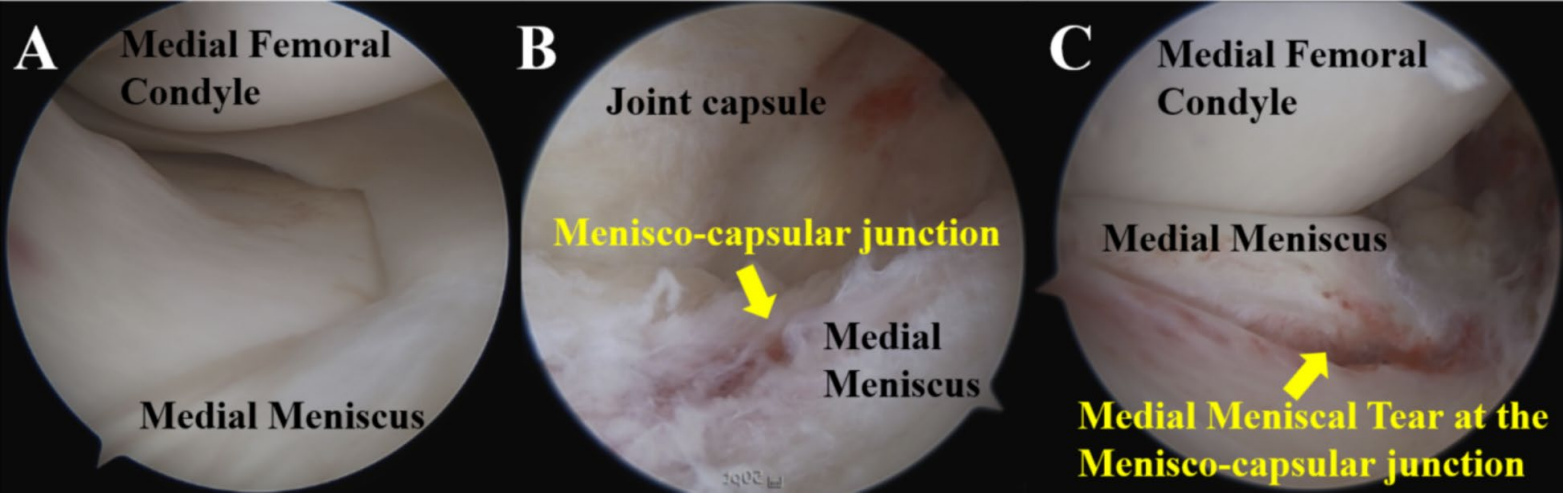
Detection of meniscal tears based on viewing portal **A** The medial meniscus appears normal when observed using a 30-degree scope with the anterolateral portal as the viewing portal. **B** Blood pigmentation is visible at the medial menisco-capsular junction using a 30-degree scope in the intercondylar notch view, but no clear meniscal tear is identified. **C** A tear at the menisco-capsular junction is clearly observed when using the posteromedial portal as the viewing portal. Even if a meniscal tear is located in Zones A0–A1 or F0–F1, it may be missed when using a 30-degree scope in the intercondylar notch view. To avoid overlooking menisco-capsular junction tears, a 70-degree scope in the intercondylar notch view or the posteromedial or posterolateral portal as the viewing portal should be used, as the tear site may be located out of a visual field of a 30-degree scope

The longitudinal tear at the menisco-capsular junction was identified as the most frequently observed meniscal injury in acute ACL ruptures. Our results demonstrated a higher incidence compared with previous studies [16, 21, 24–26], supporting our hypothesis that meniscal tears have been underestimated in earlier reports. This discrepancy is likely attributable to the meticulous evaluation of the posterior joint space, thereby enabling identification of menisco-capsular junction tears. Similar findings were reported by Sonnery-Cottet et al., who recently noted a detection rate of ramp lesions of up to 40%, aligning with our results [18, 28]. The predominant location for tears in acute ACL ruptures is the menisco-capsular junction. Therefore, relying solely on anterolateral and anteromedial portals for diagnostic arthroscopic examination without inspecting the posterior menisco-capsular junction increases the likelihood of missing these tears, as suggested by several authors [18, 28]. We recommend using a 70-degree arthroscope through the intercondylar notch or utilizing posteromedial or posterolateral portals as viewing portals. Even if the posterior joint space is evaluated using a 30-degree arthroscope passed through the intercondylar notch, caution is required, as menisco-capsular junction tears in Cooper’s classification Zone 1 around the femoral condyle may be missed owing to the limited arthroscopic view (Fig. 7).

In addition, arthroscopic evaluation of the menisco-capsular junction should be performed regardless of MRI findings. Although MRI is considered the most useful tool for detecting meniscal tears associated with ACL injury, previous meta-analyses have reported a sensitivity of approximately 71% for detecting ramp lesions [17]. In contrast, the sensitivity for detecting longitudinal tears in the present study was only 34–37.7%, showing a considerable discrepancy from previous reports.

The cause of this discrepancy remains unclear; however, it may be related to differences in the timing of arthroscopic evaluations among studies included in the meta-analysis. The mean surgical timing in those studies was approximately 6 weeks after injury. Because tears at the menisco-capsular junction are typically located in the red-red zone, which has a high healing potential, self-healing during this period may have reduced the observed incidence. In the present study, only patients with acute injuries (mean interval from trauma, about 3 weeks) were included, which could partly explain the higher incidence of ramp lesions compared with previous meta-analyses. Differences in surgical technique may also have contributed to this discrepancy. In the present study, arthroscopic inspection of the menisco-capsular junction was performed in all patients, irrespective of MRI findings. This comprehensive inspection likely increased the detection rate of longitudinal tears on arthroscopy, while MRI detection remained unchanged, thereby producing a larger difference in incidence compared with previous literature.

These findings suggest that, during acute ACL reconstruction, relying solely on MRI may result in missed detection of longitudinal tears at the menisco-capsular junction in approximately 63–66% of cases. In contrast, lateral meniscus root or radial tears are less likely to be missed, as they can be visualized through the anterior viewing portal. However, because of the low MRI sensitivity for menisco-capsular junction tears, direct arthroscopic visualization of the posterior compartment should be routinely performed, either by advancing the arthroscope through the intercondylar notch using a 70° scope or by observing the compartment directly through the posteromedial or posterolateral portals. This supports previous findings that a meticulous posterior arthroscopic examination remains the gold standard for diagnosing ramp lesions [18]. Moreover, recent studies have reported that bone bruising in the posteromedial tibial plateau on MRI is frequently associated with ramp lesions [26, 29, 30]. Therefore, when such bone marrow edema is observed, careful inspection of the posteromedial menisco-capsular junction should be performed to avoid missing an occult ramp lesion.

While the necessity of aggressive repair at the menisco-capsular junction remains controversial [31, 32], there is growing recognition that anterolateral rotatory instability following ACL rupture is associated with soft tissue structures that function as secondary stabilizers [5]. Among these, ramp lesions have been increasingly identified as key secondary stabilizers in several studies [7, 9, 10, 12, 14]. Biomechanical studies have reported a correlation between anterior tibial translation and ramp lesions [8, 9, 11, 14, 33], while some studies have also demonstrated an association between ramp lesions and rotational instability [7, 9, 10, 12, 14, 34]. In addition, ramp lesions have been linked to the progression of medial compartment osteoarthritis [35]. During ACL reconstruction, simultaneous medial meniscectomy has been reported to be associated with residual anteroposterior or rotational instability, as well as an increased failure rate of ACL reconstructions [13, 36]. These findings underscore the crucial role of the medial meniscus as a secondary stabilizer, reinforcing the growing support for preserving the meniscus as much as possible during ACL reconstruction. It remains unclear whether ramp lesions occur secondarily from chronic rotational instability caused by ACL deficiency or primarily from femoral posterolateral subluxation or a counter-coup mechanism. This study analyzed patients with relatively short durations of ACL deficiency, suggesting that ramp lesions are highly likely to occur concurrently with the initial ACL rupture. This finding implies that ACL rupture should not be viewed merely as a ligamentous injury but rather as a complex injury that induces concurrent soft tissue damage [24]. Further research is needed to determine whether chronic ACL deficiency exacerbates the incidence of ramp lesions.

**Fig. 8:**
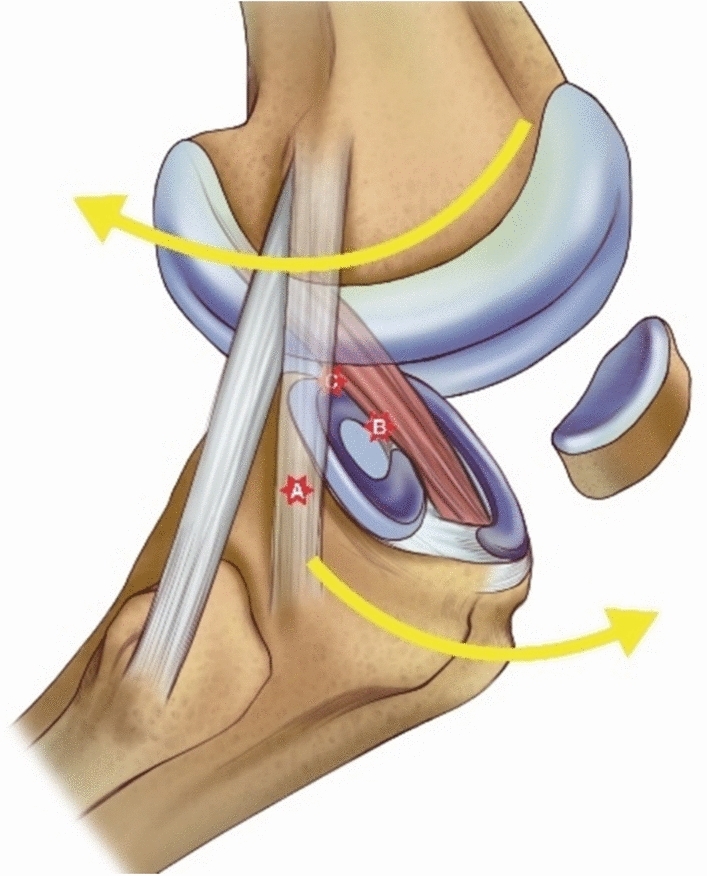
Soft-tissue structures in the lateral compartment that can be injured during pivoting motion in anterior cruciate ligament (ACL) rupture. During ACL rupture, the femur translates posterolaterally relative to the tibia. **A** The anterolateral ligament (ALL) may be injured in this process. **B** Depending on the degree of knee flexion at the time of injury, if valgus position with a dominant axial force is present, the femur rotates posterolaterally, causing a root tear of the lateral meniscus at the inner rim (corresponding to Zone 3 of Cooper’s classification). This injury may extend into Zones 2, 1, or 0 depending on the magnitude of the external rotation force. **C** Conversely, if external rotation of the femur acts as the dominant force rather than the axial load, or if the femur has already passed posterior to the lateral meniscus root at the time of injury, a tear can occur at the menisco-capsular junction of the lateral meniscus, as demonstrated in the present study

There are few reports on menisco-capsular junction tears occurring in the lateral meniscus. However, our study revealed an incidence similar to that of ramp lesions in patients with acute ACL ruptures. Although the mechanism underlying this type of tear remains uncertain, it is speculated to result from soft tissue injury in the posterolateral space, caused by rotational forces when the femur subluxates posterolaterally relative to the tibia during pivot shift motion [12, 37, 38]. An interesting finding was that the incidence of longitudinal tears at the lateral menisco-capsular junction was significantly reduced to 19% in patients with lateral meniscus root tears, compared with 44.1% in patients without lateral meniscus root tears. Considering that ACL injury mechanisms involve valgus force, axial force, and tibial internal rotation [39], this difference may be explained by variations in knee position and injury mechanisms at the time of ACL rupture, which may influence the relative dominance of each force acting on the knee. When axial force predominates over rotational force, lateral meniscus root tears may occur. Conversely, when rotational force predominates, longitudinal tears may occur at the menisco-capsular junction of the lateral meniscus (Fig. 8). Although further clinical studies are needed to determine the impact of aggressive repair on residual anterolateral rotational instability, recent studies suggest that both types of lateral meniscus tears may contribute to anterolateral rotational instability [15, 40].

A major strength of this study lies in its evaluation of patients who underwent ACL reconstruction within 6 weeks of injury, allowing for a precise determination of the location and incidence of meniscal injuries occurring concurrently with the initial ACL tear. Unlike previous studies, this research meticulously assessed the posterior menisco-capsular junction in all patients through posterior arthroscopic evaluation, revealing a higher incidence of injuries in this region than previously reported. The most significant finding of this study is that approximately 75% of acute ACL ruptures should be considered combined injuries, involving not only the ACL but also meniscal tears, with 65% of these affecting the posterior menisco-capsular junction.

However, this study has certain limitations. One potential limitation is the possible inclusion of cases with partial ACL tears. Given that 10–27% of ACL tears are partial and are associated with a relatively lower incidence of concurrent meniscal injuries [41, 42], the actual incidence of meniscal tears in acute complete ACL ruptures may have been underestimated, despite the higher overall incidence of meniscal injuries reported in our study. Another limitation is the relatively small sample size compared with previous studies, which may lead to differences when compared with larger cohort analyses. To validate our findings, future research involving larger-scale studies or multi-center trials is necessary. Lastly, as preoperative anterior drawer test results and intraoperative pivot-shift grades were not assessed, the influence of meniscal injury on anterior and rotational instability could not be evaluated.

## Conclusions

Longitudinal tears at the menisco-capsular junction are the most tear pattern in acute ACL injuries, with a higher incidence than previously reported. In acute ACL injuries, Zones A0–A1 and F0–F1 should be meticulously assessed using a 70-degree scope in the intercondylar notch view or through the posteromedial and posterolateral portals as viewing portals.

## Data Availability

The datasets generated and/or analyzed during the current study are available from the corresponding author on reasonable request.

## References

[1] Wang LJ, Zeng N, Yan ZP, Li JT, Ni GX (2020) Post-traumatic osteoarthritis following ACL injury. Arthritis Res Ther 22:57 32209130 10.1186/s13075-020-02156-5 PMC7092615

[2] Petersen W, Guenther D, Imhoff AB, Herbort M, Stein T, Schoepp C, Akoto R, Hoher J, Scheffler S, Stoehr A, Stoffels T, Haner M, Hees T, Mehl J, Ellermann A, Krause M, Mengis N, Eberle C, Muller PE, Best R, Lutz PM, Achtnich A (2023) Management after acute rupture of the anterior cruciate ligament (ACL). Part 1: ACL reconstruction has a protective effect on secondary meniscus and cartilage lesions. Knee Surg Sports Traumatol Arthrosc 31:1665–1674 35445329 10.1007/s00167-022-06960-1 PMC10089999

[3] Jonsson H, Riklund-Ahlstrom K, Lind J (2004) Positive pivot shift after ACL reconstruction predicts later osteoarthrosis: 63 patients followed 5-9 years after surgery. Acta Orthop Scand 75:594–599 15513493 10.1080/00016470410001484

[4] Mao Y, Zhang K, Li J, Fu W (2021) Supplementary lateral extra-articular tenodesis for residual anterolateral rotatory instability in patients undergoing single-bundle anterior cruciate ligament reconstruction: a meta-analysis of randomized controlled trials. Orthop J Sports Med 9:23259671211002280 33997075 10.1177/23259671211002282 PMC8113943

[5] Vaudreuil NJ, Rothrauff BB, de Sa D, Musahl V (2019) The pivot shift: current experimental methodology and clinical utility for anterior cruciate ligament rupture and associated injury. Curr Rev Musculoskelet Med 12:41–49 30706283 10.1007/s12178-019-09529-7 PMC6388573

[6] Rodriguez AN, LaPrade RF, Geeslin AG (2022) Combined meniscus repair and anterior cruciate ligament reconstruction. Arthroscopy 38:670–672 35248223 10.1016/j.arthro.2022.01.003

[7] Kato J, Fukushima H, Kensaku A, Hanaki S, Ota K, Kawanishi Y, Kobayashi M, Yoshida M, Takenaga T, Kawaguchi Y, Kuroyanagi G, Sakai H, Murakami H, Nozaki M (2025) Multiple secondary stabiliser injuries increase rotational instability in anterior cruciate ligament-deficient knees. Knee Surg Sports Traumatol Arthrosc. 10.1002/ksa.12565 39756019 10.1002/ksa.12565

[8] Ahn JH, Bae TS, Kang KS, Kang SY, Lee SH (2011) Longitudinal tear of the medial meniscus posterior horn in the anterior cruciate ligament-deficient knee significantly influences anterior stability. Am J Sports Med 39:2187–2193 21828365 10.1177/0363546511416597

[9] DePhillipo NN, Moatshe G, Brady A, Chahla J, Aman ZS, Dornan GJ, Nakama GY, Engebretsen L, LaPrade RF (2018) Effect of meniscocapsular and meniscotibial lesions in ACL-deficient and ACL-reconstructed knees: a biomechanical study. Am J Sports Med 46:2422–2431 29847148 10.1177/0363546518774315

[10] Mouton C, Magosch A, Pape D, Hoffmann A, Nuhrenborger C, Seil R (2020) Ramp lesions of the medial meniscus are associated with a higher grade of dynamic rotatory laxity in ACL-injured patients in comparison to patients with an isolated injury. Knee Surg Sports Traumatol Arthrosc 28:1023–1028 31250053 10.1007/s00167-019-05579-z

[11] Stephen JM, Halewood C, Kittl C, Bollen SR, Williams A, Amis AA (2016) Posteromedial meniscocapsular lesions increase tibiofemoral joint laxity with anterior cruciate ligament deficiency, and their repair reduces laxity. Am J Sports Med 44:400–408 26657852 10.1177/0363546515617454

[12] Kim YS, Koo S, Kim JH, Tae J, Wang JH, Ahn JH, Jang KM, Jeon J, Lee DK (2023) Greater knee rotatory instability after posterior meniscocapsular injury versus anterolateral ligament injury: a proposed mechanism of high-grade pivot shift. Orthop J Sports Med 11:23259671231188710 37693803 10.1177/23259671231188712 PMC10486219

[13] Jacquet C, Pioger C, Seil R, Khakha R, Parratte S, Steltzlen C, Argenson JN, Pujol N, Ollivier M (2021) Incidence and risk factors for residual high-grade pivot shift after ACL reconstruction with or without a lateral extra-articular tenodesis. Orthop J Sports Med 9:23259671211003590 33997078 10.1177/23259671211003590 PMC8113945

[14] Hanaki S, Fukushima H, Abe K, Ota K, Kobayashi M, Kawanishi Y, Kato J, Yoshida M, Takenaga T, Murakami H, Nozaki M (2024) Greater rotational knee laxity observed at second-look arthroscopy in patients with failed meniscal repair performed at the time of anterior cruciate ligament reconstruction. Arthroscopy. 10.1016/j.arthro.2024.12.021 39725048 10.1016/j.arthro.2024.12.021

[15] Katakura M, Horie M, Watanabe T, Katagiri H, Otabe K, Ohara T, Nakamura K, Katagiri K, Ueki H, Zaffagnini S, Sekiya I, Muneta T, Koga H (2019) Effect of meniscus repair on pivot-shift during anterior cruciate ligament reconstruction: objective evaluation using triaxial accelerometer. Knee 26:124–131 30554908 10.1016/j.knee.2018.11.016

[16] Seil R, Mouton C, Coquay J, Hoffmann A, Nuhrenborger C, Pape D, Theisen D (2018) Ramp lesions associated with ACL injuries are more likely to be present in contact injuries and complete ACL tears. Knee Surg Sports Traumatol Arthrosc 26:1080–1085 28638970 10.1007/s00167-017-4598-3

[17] Koo B, Lee SH, Yun SJ, Song JG (2020) Diagnostic performance of magnetic resonance imaging for detecting meniscal ramp lesions in patients with anterior cruciate ligament tears: a systematic review and meta-analysis. Am J Sports Med 48:2051–2059 31684739 10.1177/0363546519880528

[18] Sonnery-Cottet B, Conteduca J, Thaunat M, Gunepin FX, Seil R (2014) Hidden lesions of the posterior horn of the medial meniscus: a systematic arthroscopic exploration of the concealed portion of the knee. Am J Sports Med 42:921–926 24567252 10.1177/0363546514522394

[19] Slauterbeck JR, Kousa P, Clifton BC, Naud S, Tourville TW, Johnson RJ, Beynnon BD (2009) Geographic mapping of meniscus and cartilage lesions associated with anterior cruciate ligament injuries. J Bone Joint Surg Am 91:2094–2103 19723985 10.2106/JBJS.H.00888 PMC7002077

[20] Smith JP, Barrett GR (2001) Medial and lateral meniscal tear patterns in anterior cruciate ligament-deficient knees. A prospective analysis of 575 tears. Am J Sports Med 29:415–9 11476378 10.1177/03635465010290040501

[21] Keyhani S, Esmailiejah AA, Mirhoseini MS, Hosseininejad SM, Ghanbari N (2020) The prevalence, zone, and type of the meniscus tear in patients with anterior cruciate ligament (ACL) injury; does delayed ACL reconstruction affects the meniscal injury? Arch Bone Jt Surg 8:432–438 32766404 10.22038/abjs.2019.39084.2076 PMC7358246

[22] Michalitsis S, Vlychou M, Malizos KN, Thriskos P, Hantes ME (2015) Meniscal and articular cartilage lesions in the anterior cruciate ligament-deficient knee: correlation between time from injury and knee scores. Knee Surg Sports Traumatol Arthrosc 23:232–239 23595538 10.1007/s00167-013-2497-9

[23] Hagino T, Ochiai S, Senga S, Yamashita T, Wako M, Ando T, Haro H (2015) Meniscal tears associated with anterior cruciate ligament injury. Arch Orthop Trauma Surg 135:1701–1706 26286641 10.1007/s00402-015-2309-4

[24] Gracia G, Cavaignac M, Marot V, Mouarbes D, Laumonerie P, Cavaignac E (2022) Epidemiology of combined injuries of the secondary stabilizers in ACL-deficient knees: medial meniscal ramp lesion, lateral meniscus root tear, and ALL tear: a prospective case series of 602 patients with ACL tears from the SANTI study group. Am J Sports Med 50:1843–1849 35416066 10.1177/03635465221092767

[25] Liu X, Feng H, Zhang H, Hong L, Wang XS, Zhang J (2011) Arthroscopic prevalence of ramp lesion in 868 patients with anterior cruciate ligament injury. Am J Sports Med 39:832–837 21220541 10.1177/0363546510388933

[26] DePhillipo NN, Cinque ME, Chahla J, Geeslin AG, Engebretsen L, LaPrade RF (2017) Incidence and detection of meniscal ramp lesions on magnetic resonance imaging in patients with anterior cruciate ligament reconstruction. Am J Sports Med 45:2233–2237 28463534 10.1177/0363546517704426

[27] El Helou A, Gousopoulos L, Shatrov J, Hopper GP, Philippe C, Ayata M, Thaunat M, Fayard JM, Freychet B, Vieira TD, Sonnery-Cottet B (2023) Failure rates of repaired bucket-handle tears of the medial meniscus concomitant with anterior cruciate ligament reconstruction: a cohort study of 253 patients from the SANTI study group with a mean follow-up of 94 months. Am J Sports Med 51:585–595 36734511 10.1177/03635465221148497

[28] Kim SH, Lee SH, Kim KI, Yang JW (2018) Diagnostic accuracy of sequential arthroscopic approach for ramp lesions of the posterior horn of the medial meniscus in anterior cruciate ligament-deficient knee. Arthroscopy 34:1582–1589 29402583 10.1016/j.arthro.2017.12.008

[29] Green JS, Moran J, Marcel A, Joo PY, McLaughlin WM, Manzi JE, Yalcin S, Wang A, Porrino J, Jimenez AE, Medvecky MJ, Katz LD (2023) Posteromedial tibial plateau bone bruises are associated with medial meniscal ramp lesions in patients with concomitant anterior cruciate ligament ruptures: a systematic review and meta-analysis. Phys Sportsmed 51:531–538 35915996 10.1080/00913847.2022.2108350

[30] Beel W, Mouton C, Tradati D, Nuhrenborger C, Seil R (2022) Ramp lesions are six times more likely to be observed in the presence of a posterior medial tibial bone bruise in ACL-injured patients. Knee Surg Sports Traumatol Arthrosc 30:184–191 33661324 10.1007/s00167-021-06520-z

[31] Saint-Etienne A, Benhenneda R, Vieira TD, Fayard JM, Thaunat M (2024) Clinical outcomes of different management techniques for medial meniscal type 3 ramp lesions in anterior cruciate ligament reconstruction: a comparative analysis between all-inside repair, suture hook repair, and lesions left in situ. Am J Sports Med 52:1250–1257 38523481 10.1177/03635465241232088

[32] Tanel L, Thaunat M, Lambrey PJ, Portet A, Vincent A, Vieira TD, Jan N, Fayard JM (2024) Survivorship and outcomes of meniscal ramp lesions repaired through a posteromedial portal during anterior cruciate ligament reconstruction: outcome study with a minimum 10-year follow-up. Am J Sports Med 52:3206–3211 39439282 10.1177/03635465241288233

[33] Peltier A, Lording T, Maubisson L, Ballis R, Neyret P, Lustig S (2015) The role of the meniscotibial ligament in posteromedial rotational knee stability. Knee Surg Sports Traumatol Arthrosc 23:2967–2973 26264383 10.1007/s00167-015-3751-0

[34] Kawanishi Y, Kobayashi M, Yasuma S, Fukushima H, Kato J, Murase A, Takenaga T, Yoshida M, Kuroyanagi G, Kawaguchi Y, Murakami H, Nozaki M (2024) Factors associated with residual pivot shift after ACL reconstruction: a quantitative evaluation of the pivot-shift test preoperatively and at minimum 12-month follow-up. Orthop J Sports Med 12:23259671241230970 38414663 10.1177/23259671241230967 PMC10898316

[35] Guimaraes JB, Schwaiger BJ, Gersing AS, Neumann J, Facchetti L, Li X, Joseph GB, Link TM (2021) Meniscal ramp lesions: frequency, natural history, and the effect on knee cartilage over 2 years in subjects with anterior cruciate ligament tears. Skeletal Radiol 50:551–558 32901305 10.1007/s00256-020-03596-5 PMC7854891

[36] Roe J, Pinczewski LA, Russell VJ, Salmon LJ, Kawamata T, Chew M (2005) A 7-year follow-up of patellar tendon and hamstring tendon grafts for arthroscopic anterior cruciate ligament reconstruction: differences and similarities. Am J Sports Med 33:1337–1345 16002487 10.1177/0363546504274145

[37] Pringle L, Wissman RD (2022) Imaging of noncontact anterior cruciate ligament injuries and associated bone marrow contusion patterns. J Knee Surg 35:475–481 34902869 10.1055/s-0041-1740393

[38] Zhang L, Hacke JD, Garrett WE, Liu H, Yu B (2019) Bone bruises associated with anterior cruciate ligament injury as indicators of injury mechanism: a systematic review. Sports Med 49:453–462 30689129 10.1007/s40279-019-01060-6

[39] Kiapour AM, Demetropoulos CK, Kiapour A, Quatman CE, Wordeman SC, Goel VK, Hewett TE (2016) Strain response of the Anterior Cruciate Ligament to uniplanar and multiplanar loads during simulated landings: implications for injury mechanism. Am J Sports Med 44:2087–2096 27159285 10.1177/0363546516640499

[40] Hoshino Y, Hiroshima Y, Miyaji N, Nagai K, Araki D, Kanzaki N, Kakutani K, Matsushita T, Kuroda R (2020) Unrepaired lateral meniscus tears lead to remaining pivot-shift in ACL-reconstructed knees. Knee Surg Sports Traumatol Arthrosc 28:3504–3510 32328696 10.1007/s00167-020-06007-3

[41] Stone AV, Marx S, Conley CW (2021) Management of partial tears of the Anterior Cruciate Ligament: a review of the anatomy, diagnosis, and treatment. J Am Acad Orthop Surg 29:60–70 33394613 10.5435/JAAOS-D-20-00242

[42] Gupta R, Singhal A, Sharma AR, Shail S, Masih GD (2021) Strong association of meniscus tears with complete Anterior Cruciate Ligament (ACL) injuries relative to partial ACL injuries. J Clin Orthop Trauma 23:101671 34790561 10.1016/j.jcot.2021.101671 PMC8577485

